# Sudden unexplained death in alcohol misuse (SUDAM) patients have different characteristics to those who died from sudden arrhythmic death syndrome (SADS)

**DOI:** 10.1007/s12024-017-9877-2

**Published:** 2017-07-01

**Authors:** Tracy Sorkin, Mary N. Sheppard

**Affiliations:** 1 St George’s Healthcare NHS Trust, Cellular Pathology, St George’s Hospital, Blackshaw Road, London, SW17 0QT UK; 2 CRY Department of Cardiovascular Pathology, St George’s, University of London, Cranmer Terrace, London, SW17 0RE UK

**Keywords:** SUDAM, SADS, Alcohol, Sudden death, Cardiac

## Abstract

There is growing awareness of sudden unexplained death in alcohol misuse (SUDAM) in which there is no obvious cause of death, no evidence of acute alcohol toxicity or alcoholic ketoacidosis, and the heart is morphologically normal. This study describes the characteristics of a cohort with SUDAM from a tertiary cardiovascular referral center and compares the findings with those of individuals who died from sudden arrhythmic death syndrome (SADS). Cases in this retrospective cross-sectional study were identified from a database of referrals to our center spanning approximately 40 years. Cases with recorded heavy use of alcohol and non-alcohol users were selected, then limited to those with SUDAM or SADS aged 16 to 64 years. 62 cases of SUDAM and 41 cases of SADS were identified. The SUDAM group were older than the SADS group; mean age 35.8 years and 27.7 years respectively (P=0.0002). There was also a higher incidence of significant psychiatric illness in SUDAM (19.7%) than SADS (2.4%) cases. Post mortem liver examination was more likely to reveal heavy livers in SUDAM than SADS (2196.1g and 1572.4g respectively; P=0.0033) and more fatty liver change (24.2% and 2.4%). SUDAM tends to occur in individuals who are older and have heavier livers than those with SADS. Psychiatric illness is also more common. SADS, unlike SUDAM, is often associated with heritable channelopathies that may affect surviving family members. Therefore, differentiating between SUDAM and SADS identifies families likely to benefit from screening for these mutations, thus preventing further sudden arrhythmic deaths.

## Introduction

Alcohol misuse accounts for 1.4% of deaths in England and Wales and is associated with a greater risk of sudden death than the general population [1–4]. Causes of death in heavy alcohol use are related to trauma, acute intoxication or alcohol-related disease such as cirrhosis [5]. The cause of death may be obvious at autopsy, such as ischemic heart disease or pneumonia, but when the only finding at autopsy is a fatty liver the investigation requires comprehensive histological, toxicological and biochemical assessment.

Case series have described heavy alcohol users dying suddenly with the only abnormality at autopsy examination being fatty liver change. A study of such cases in Tokyo identified the deaths of 534 chronic alcoholics, 226 (42%) were designated ‘alcohol-related sudden death with hepatic fatty metamorphosis (ASDHFM)’ [6]. Further study of a subset of 11 such cases of ASDHFM in people who died in hospital following initial resuscitation showed they had profound hypoglycemia and metabolic acidosis [7]. In these cases, toxicological causes of death were excluded but alcoholic ketoacidosis was not. Templeton et al. subsequently identified 162 deaths related to alcohol which had post mortem (PM) examinations in Southampton. Causes of death in four cases had no morphological explanation of death and were negative for acute toxicity and ketoacidosis [8]. They proposed the entity sudden unexplained death in alcohol misuse (SUDAM) as a cause for these deaths and suggested the definition: “sudden, unexpected, unwitnessed or witnessed, non-traumatic deaths in patients with a history of chronic excess alcohol consumption and or evidence of hepatic steatosis or other alcoholic liver disease where PM examination does not reveal a toxicological (specifically alcohol intoxication or alcoholic ketoacidosis are excluded) or anatomical cause of death and there is no significant cardiac hypertrophy”.

Death due to sudden arrhythmic death syndrome (SADS) may have repercussions for surviving family members. The cause of SADS is often a channelopathy, and in many instances these are heritable; in one study 22% of families were diagnosed with inherited cardiac disease following the death of the proband from SADS [9]. Identifying these affected family members allows them to receive appropriate management, such as implantable cardiac defibrillators, thus reducing the likelihood of these people also dying from fatal arrhythmias.

There are no published studies comparing deaths attributed to SUDAM with those attributed to SADS. This study will describe the characteristics of people who meet the criteria for SUDAM and compare them to non-alcohol drinkers diagnosed with SADS in order to explore potential discriminatory factors between the two groups. Being able to better discriminate between these two diagnoses of exclusion in heavy alcohol users may be useful in initiating screening of family members for potentially fatal ion channelopathies.

## Method

Cases in this cross-sectional study were identified by retrospective review of the database at our specialist cardiac pathology referral center; cases date from 1971 to 2014. All non-alcohol drinkers and heavy alcohol users, aged 16–64 years with normal toxicology results, whose deaths were diagnosed as SADS or SUDAM were selected. People with heavy alcohol use were those reported to have a significant chronic alcohol history by a medical practitioner, either their general practitioner (GP) or the referring pathologist. Heavy alcohol use is typically defined as “binge drinking on five or more days in the past month” when binge drinking is imbibing four to five drinks (of 14 g pure alcohol) in approximately two hours [10]. The diagnosis of SUDAM was made in line with the parameters described by Templeton and colleagues; a history of chronic excess alcohol consumption, no significant cardiac pathology, no anatomical cause of death, no toxicological cause of death, and no evidence of ketoacidosis on biochemical investigation [8]. In order to avoid potential confounding factors cases with a history of epilepsy, anorexia, or diabetes were excluded as these conditions have also been associated with sudden unexplained deaths [11–13].

Data analysis was performed using a standard spreadsheet package (Microsoft Excel 2010). Statistical analysis (unpaired t-tests) was performed using GraphPad Prism (V6.01 2012). STROBE guidance was followed throughout the course of this study.

## Results

The population identified comprised 62 heavy alcohol users whose deaths matched criteria for SUDAM, and 41 non-drinkers whose deaths were attributed to SADS. Comparison of demographic parameters revealed the SUDAM group to be older (*P* = 0.0002); mean age 35.8 years, compared with 27.7 years for SADS (Table 1). The male to female ratios were 46:16 in SUDAM and 27:14 SADS (Table 1). The predominant ethnic group was white British in both SUDAM (48, 77.4%) and SADS (25, 61.0%; Table 1). There was no statistically significant difference in body mass index (BMI) between the two groups (*P* = 0.4245; Table 2).

**Table 1:** Comparison of demographic parameters for heavy alcohol users who died from SUDAM and non-alcohol users who died from SADS

Demographic Parameters		SUDAM (*n* = 62)	SADS (*n* = 41)
Age (years)	Mean ± SD	35.8 ± 9.8*	27.7 ± 11.2*
	Range	18–59	16–58
Gender	Female	16 (25.8%)	14 (34.1%)
	Male	46 (74.2%)	27 (65.9%)
Ethnicity	White (British)	48 (77.4%)	25 (61.0%)
	White (Irish)	1 (1.6%)	1 (2.4%)
	White (Other)	1 (1.6%)	1 (2.4%)
	Black African	1 (1.6%)	2 (4.9%)
	Black Caribbean	0	2 (4.9%)
	Indian	0	2 (4.9%)
	Other Asian	0	2 (4.9%)
	Unknown	11 (17.7%)	6 (14.6%)

*SUDAM* Sudden unexpected death in alcohol misuse

*SADS* Sudden adult/arrhythmic death syndrome

*SD* Standard deviation

*There was a statistically significant difference (*P* = 0.0002)

**Table 2:** Comparison of BMI for heavy alcohol users who died from SUDAM and non-alcohol users who died from SADS

BMI (kg/m^2^)	SUDAM (*n* = 62)	SADS (*n* = 41)
Mean ± SD	24.1 ± 7.4	25.2 ± 10.6
Range	10.3–38.3	18.4–38.9

There was no statistically significant difference in the BMI of SUDAM and SADS (*P* = 0.4245).

*BMI* Body mass index

*SUDAM* Sudden unexpected death in alcohol misuse

*SADS* Sudden adult/arrhythmic death syndrome

*SD* Standard deviation

The recorded co-existing medical conditions were broadly similar for the SUDAM and SADS groups (Table 3). However, significant psychiatric illness including major depressive illness and schizophrenia were more common in the heavy alcohol users; 12 cases (19.4%) compared to 1 case (2.4%) for SADS. People who died from SUDAM were also more likely to be known illicit drug users than those who died from SADS (25.8% and 0% respectively; Table 3). There was no established history regarding illicit drug use in 43 (69.4%) cases of SUDAM and 12 (29.3%) cases of SADS.

**Table 3:** Comparison of significant co-existing conditions for heavy alcohol users who died from SUDAM and non-alcohol users who died from SADS

Significant co-existing conditions	SUDAM (*n* = 62)	SADS (*n* = 41)
Illicit drug use	16 (25.8%)	0
Significant psychiatric illness	12 (19.4%)	1 (2.4%)
Asthma	4 (6.5%)	4 (9.8%)
Alcohol-related liver disease	4 (6.5%)	0
Pregnant/post-partum	3 (4.8%)	1 (2.4%)
Congenital heart disease	1 (1.6%)	0
Neuromuscular disease	1 (1.6%)	0
Athlete	0	1 (2.4%)

*SUDAM* Sudden unexpected death in alcohol misuse

*SADS* Sudden adult/arrhythmic death syndrome

The circumstances of death were similar between the two groups, with the majority of people dying at rest or during sleep: SUDAM 49 (79.0%) and SADS 34 (82.9%; Table 4). The circumstances were unknown for more cases in the SUDAM (8, 12.9%) group than SADS (1, 2.4%). It was not possible to reliably compare the occurrence of family history of sudden death in people who died from SUDAM and SADS as the data was missing in 51 SUDAM cases (82.2%) and 29 SADS cases (68.3%). It is proposed that the reason for limited information regarding the circumstances surrounding death and family history in the SUDAM group is a result of social isolation which is often associated with heavy alcohol use.

**Table 4:** Comparison of circumstances of death for heavy alcohol users who died from SUDAM and non-alcohol users who died from SADS

Circumstances of death	SUDAM (*n* = 62)	SADS (*n* = 41)
Died at rest	34 (54.8%)	22 (53.7%)
Died in sleep	15 (24.2%)	12 (29.3%)
Died during exertion	2 (3.2%)	6 (14.6%)
Died during emotional stress	3 (4.8%)	0
Circumstances unknown	8 (12.9%)	1 (2.4%)

*SUDAM* Sudden unexpected death in alcohol misuse

*SADS* Sudden adult/arrhythmic death syndrome

PM liver examination descriptions were available for 39 (62.9%) SUDAM cases and 16 (38.1%) SADS cases (Table 5). Reported liver weights of SUDAM cases were significantly greater than for SADS cases; mean weights 2196 g and 1572 g respectively (*P* = 0.0033). Notably more fatty livers were encountered in the SUDAM group (15, 24.2%) compared with SADS (1, 2.4%). Liver microscopy results were not frequently reported; this reflects the limited taking of histology in coronial autopsy examinations.

**Table 5:** Comparison of post mortem liver examination findings for heavy alcohol users who died from SUDAM and non-alcohol users who died from SADS

Post mortem liver description		SUDAM (*n* = 62)	SADS (*n* = 41)
Liver weight (g)	Weight available	12	7
	Mean ± SD (g)	2196.1 ± 343.3*	1572.4 ± 450.6*
	Range (g)	1596-3000 g	1000–2401
Morphological Findings	Normal	21 (33.9%)	11 (26.8%)
	Congestion	2 (3.2%)	4 (9.8%)
	Fatty	15 (24.2%)**	1 (2.4%)
	Cirrhosis	1 (1.6%)	0
	Unknown	23 (37.1%)	25 (61.0%)

*SUDAM* Sudden unexpected death in alcohol misuse

*SADS* Sudden adult/arrhythmic death syndrome

*SD* Standard deviation

*There was a statistically significant difference (*P* = 0.0033)

**Including one case of microscopically confirmed steatohepatitis.

Differences in specific cardiac parameters and toxicology/biochemistry results were not explored, as these were all essentially normal in order to meet the diagnostic criteria of SUDAM and SADS.

## Discussion

Labelled in 2009 as sudden death in alcohol misuse, SUDAM has been increasingly recognized as a cause of death [8]. Our study identified 62 cases which meet the definition of SUDAM; a larger case series than previously reported. This is the first series to compare those dying from SUDAM with patients dying suddenly with negative autopsy and with no alcohol use (SADS). Although SUDAM and SADS are diagnoses of exclusion it is important to differentiate between these two groups, on the balance of probabilities, as it can have significant implications for surviving family members. SADS is associated with heritable channelopathies and screening of family members is advised and in some circumstances funding for this can be covered by charitable organizations. Screening of family members has revealed such channelopathies in one fifth of cases, allowing appropriate steps to be taken to reduce their risk of sudden arrhythmic death, such as through careful monitoring and the use of implantable cardiac defibrillators [9]. No such association with channelopathies has been identified in SUDAM and there are no current recommendations for seeking such mutations.

Studies of sudden nonviolent deaths in alcoholics illustrate the largely unrecognized and frequent occurrence of sudden death with autopsy findings limited solely to fatty liver i.e. SUDAM. The mechanism(s) of these sudden fatty liver deaths is unknown. Sudden death in alcoholism is usually seen in an older (greater than 50 years) white male who dies from “chronic alcoholism” with a terminal negative blood alcohol. This victim is usually “found dead” at home with a past history of drinking, and histopathologically the liver depicts fatty change rather than cirrhosis [14, 15]. In chronic alcoholics occurrence of hepatomegaly is associated with death at a younger age [16]. Several attractive theories attribute such deaths to ethanol withdrawal induced hypoglycemia or hypomagnesemia, pulmonary fat embolization from fatty liver, or other facets of the alcohol withdrawal syndrome, including ethanol dependent maladaptive derangements of neurotransmitters. All the theories are untested [17]. It is possible that in some cases a hitherto silent channelopathy becomes unmasked by persistent heavy alcohol use. Thus, family members may benefit from screening for channelopathies in cases of SUDAM, as already advocated in SADS. However, this is an area which would benefit from further research and there is no current evidence to recommend this course of action.

Momentary intake of a large quantity of alcohol provokes ventricular ectopic activity increasing electrical instability; four out of ten of the victims of unexpected sudden cardiac death have evidence of alcohol intake before the fatal event in the Finland autopsy population [18]. There appears to be a consistent finding of an immediately higher cardiovascular risk following any alcohol consumption, but by 24 hours, only heavy alcohol intake confers continued risk [19]. Alcoholism is known to be greatly underdiagnosed in death certificates, a fact that biases estimates of alcohol-related mortality. An autopsy series of 1658 cases showed that death certificates mentioned alcohol-related diseases in less than half of these cases [20].

In comparison to the SADS group, people who died from SUDAM were significantly older and the mean liver weight was significantly greater than those who died from SADS. Liver weight does correlate with obesity; but as BMI was not significantly different between non-drinkers and heavy alcohol users it is not thought that this can account for the difference in this study [21]. The SUDAM group also showed a ten-fold greater incidence of fatty liver change than the SADS group. These liver changes, increased weight and steatosis, are recognized as changes associated with increased alcohol intake and the presence of a fatty liver has been shown to correlate with an alcohol intake of greater than 80 g (10 units) per day [22]. Although these findings are arguably anticipated, they gain significance as it has not previously been demonstrated that they are typically present in SUDAM but not SADS.

People dying from SUDAM were more likely to take illicit drugs and to have significant psychiatric illness. It is well known that psychiatric illness is associated with rates of sudden death 3–5 times greater than the general population; this may be the result of the underlying pathology or medication required for its management [23]. It has been shown that QT interval prolongation is associated with increased risk of sudden cardiac death in the context of alcoholic liver disease and psychiatric illness [23, 24].

This study, like many retrospective analyses, could have benefited from greater detail in the available data. This is particularly true of the availability of liver histology and family history of sudden cardiac death. A more quantifiable definition of heavy alcohol use would also have allowed investigation of the magnitude and duration of excess alcohol use and the post mortem changes observed in this cohort. However, it is challenging to obtain an accurate history of alcohol intake, and amongst heavy alcohol users there may be particularly poor correlation between self-reported alcohol intake and actual alcohol intake [25, 26].

The diagnosis of SUDAM, like other sudden unexplained death syndromes, is contentious and depends on getting a history of alcohol abuse. This makes research in the field of sudden unexplained death challenging. A detailed medical and social history is essential to highlight this entity. The post mortem findings of increased liver weight and steatosis, particularly in the presence of a history of psychiatric illness, could be used to prompt further investigation of chronic excess alcohol use that has not previously elicited. This would further aid differentiating between SUDAM and SADS, and subsequent recommendations for screening for channelopathy in family members.

## Conclusion

SUDAM is an increasingly recognized cause of death in heavy alcohol users. Our study shows that people who die from SUDAM tend to be older and have heavier livers which are more likely to demonstrate fatty change than people who died from SADS. People with SUDAM are also more likely to have a positive history of psychiatric illness and illicit drug use. Using these features to distinguish between people more likely to have died from SUDAM than SADS it is possible to triage the families of these individuals most likely to benefit from screening for heritable channelopathies. It is known that channelopathies occur in the families of many people who die from SADS, this has not been demonstrated in those who die from SUDAM. By identifying such mutations in family members, and initiating appropriate management, it is possible to prevent further sudden arrhythmic cardiac deaths.

## Key Points

• SUDAM and SADS are diagnoses of exclusion, distinguished by a history of persistent heavy alcohol use in those who die from SUDAM.

• In this cohort, people who died from SUDAM tended to be older, have heavier livers at PM examination, and are more likely to have a history of psychiatric illness than those who died from SADS.

• SADS, unlike SUDAM, is often associated with heritable channelopathies that may affect surviving family members. Differentiating between SUDAM and SADS identifies families likely to benefit from screening for these mutations, thus preventing further sudden arrhythmic deaths.
